# Preliminary Investigation into the Association between Scoliosis and Hypoxia: A Retrospective Cohort Study on the Impact of Eliminating Hypoxic Factors on Scoliosis Outcomes

**DOI:** 10.3390/children11091134

**Published:** 2024-09-18

**Authors:** Fatih Ugur, Kubra Topal, Mehmet Albayrak, Recep Taskin, Murat Topal

**Affiliations:** 1 Department of Orthopaedics and Traumatology, Kastamonu University Medical Faculty, 37100 Kastamonu, Turkey; rtaskin@kastamonu.edu.tr (R.T.); murattopal@kastamonu.edu.tr (M.T.); 2 Department of Otorhinolaryngology, Private Clinic, 37100 Kastamonu, Turkey; drkubratopal@gmail.com; 3 Department of Orthopaedics and Traumatology, Private Practice, 59000 Tekirdag, Turkey; doktorm.albayrak@gmail.com

**Keywords:** adenoidectomy, Cobb angle, scoliotic attitude

## Abstract

Objective: This study delves into the implications of adenoidectomy for scoliosis progression, investigating the intricate nexus of hypoxia, spinal curvature, and surgical intervention. With adenoidectomy being a common procedure for addressing pediatric sleep-disordered breathing, this research study explores its potential impact on spinal health. Patients and Methods: Employing a retrospective cohort design, this study gathered data from patients who underwent adenoidectomy, including those with scoliosis, between January 2017 and March 2023. Initial and follow-up evaluations involved clinical and radiological assessments, notably measuring the Cobb angle to quantify spinal curvature. Results: This study enrolled 218 patients under 10 years old. Among them, 18 exhibited Cobb angles of 10° or more, with a mean Cobb angle of 12.8°. In the follow-up evaluation, 83% of patients with initial Cobb angles of 10° or more were reached out to, along with 84.6% of those with Cobb angles below 10°. The postoperative follow-up revealed a notable decrease in Cobb angles for most patients, particularly those with an initial Cobb angle exceeding 10°. Conclusions: This study underscores the potential connection between adenoidectomy, hypoxia, and scoliosis regression, highlighting the importance of early intervention for scoliosis management. Despite certain limitations, this investigation lays the foundation for future research involving larger patient cohorts and multifaceted analyses. The observed interactions between airway function, hypoxia, and spinal health open avenues for refining clinical strategies in scoliosis treatment.

## 1. Introduction

Scoliosis is a common three-dimensional spinal disorder observed in both children and adults [1]. Although it is known that the prevalence of this condition varies geographically and ethnically, it ranges between 0.47% and 5.2% in the general population [2]. In 1966, the Scoliosis Research Society established the Cobb angle as the standard measure for the diagnosis and evaluation of scoliosis [3,4]. A Cobb angle of less than 10 degrees is classified as spinal curvature rather than scoliosis [3]. It has been suggested that asymmetric changes in the delicate mechanical balance of the spine during growth can lead to the development of scoliosis [5]. The risk factors influencing this include genetic, hormonal, biomechanical, neurological, and environmental factors [6,7,8]. The widely accepted theory posits that scoliosis is a multifactorial disease [9,10].

Hypoxia, identified as an environmental factor, has been implicated as one of the causes responsible for the development of congenital scoliosis [11,12]. Riverd et al. [13] demonstrated the teratogenic effects of hypoxia, which led to congenital vertebral malformations in mice. In tissues, hypoxia is regulated by the alpha subunits of the hypoxia-inducible factors, HIF-1α and HIF-2α [14,15]. Under hypoxic conditions, significant changes have been observed, particularly in intervertebral disc cells and the extracellular matrix. Additionally, it is known that hypoxia-induced alterations cause significant changes in intervertebral discs and chondrocytes, where HIF proteins are consistently maintained at high levels [15,16]. Although there is insufficient research to establish a definitive link between idiopathic scoliosis and hypoxia, Tam et al. [14] suggested that the abnormal expression of hypoxia-inducible factor (HIF) 2α, potentially influenced by hypoxia, could be a risk factor for adolescent idiopathic scoliosis (AIS) and relevant to its prognosis. Li et al. [17] indicated that elevated oxidative stress, combined with increased HIF-1α transcription due to hypoxia, may lead to apoptosis and the disruption of myogenesis in muscle cells among patients with idiopathic scoliosis.

In patients with AIS, significantly higher levels of methylation and lower expression of the PITX1 gene have been observed [18]. It is known that hypoxia leads to the downregulation of the PITX1 gene in cellular cultures of human osteoblasts. Interestingly, HIF-2 has been identified as having greater potency than HIF-1 in activating Pitx1 transcription, and partial inactivation of the Pitx1 gene results in severe spinal deformities [19].

It has been demonstrated that the effects of hypoxia can lead to irreversible changes in tissues over time [20]. Therefore, it is essential to demonstrate the impact of eliminating hypoxia on the spinal column. Adenoidectomy, frequently performed by otolaryngologists, is a procedure commonly associated with upper airway obstruction in children [21,22,23]. Postoperatively, it has been shown that the effects of hypoxia diminish and that HIF levels decrease [24,25]. In these patients, the objective is to evaluate the scoliosis values of those with scoliotic curvature after adenoidectomy and specifically assess changes in the Cobb angle.

## 2. Patients and Methods

This single-center retrospective cohort study was approved in accordance with the Declaration of Helsinki by the Kastamonu Training and Research Hospitals’ Medical Ethics Review Committee (2023-KAEK-36, date of approval: 22 March 2023). Written informed consent was obtained from the parents of all participants.

Following a retrospective analysis of chest roentgenographs in patients who underwent adenoidectomy at our institution between January 2017 and March 2023, those identified with scoliotic posture and spinal attitudes assessed by Cobb angle measurements were recalled postoperatively for orthoroentgenographic imaging, including evaluations for scoliosis. All radiologic data were obtained from the picture archiving and communication system database of the hospital.

Some chest roentgenographs, particularly in pediatric patients, may deviate from standard chest imaging and can transform into images that capture the entire spinal column instead of focusing solely on the lungs [26]. Pan et al. [27] noted in their study on the prevalence of scoliosis using roentgenographs that, as indicated in research assessing scoliosis through standard chest roentgenographs, lumbar scoliosis can be particularly overlooked [28,29].

To assess postoperative outcomes, we performed clinical examinations using Adam’s forward bending test. This information was used in conjunction with radiological data, including the Cobb angle values and the postoperative changes in Cobb angle, from patients with adequate hospital records regarding scoliosis assessment, which were available for at least 6 months following the initial evaluation.

The primary objective of the clinical assessment was to identify underlying causes and differentiate idiopathic cases through comprehensive anamnesis. The factors considered included family predisposition, existing health conditions, the presence of pain, neurological symptoms, congenital conditions, shoulder and pelvic asymmetry, and discrepancies in leg length, along with basic demographic data [30].

The Cobb angle was measured using a digital angle measurement tool. Two experienced orthopedic surgeons, who were trained in the measurement process, were then involved in reliability testing, examining how to effectively utilize the hospital’s picture archiving and communication system database, conduct digital Cobb angle measurements, and accurately record findings. Vertebral deformities in the coronal plane were assessed on plain posteroanterior radiographs by measuring the angle between the superior endplate of the uppermost tilted vertebra and the inferior endplate of the lowermost tilted vertebra [3]. This procedure was conducted for each patient with vertebral curvatures. To minimize potential measurement errors, two experienced orthopedic surgeons independently performed measurements for the initial evaluation and the last follow-up radiographs. The smallest Cobb angle value was chosen as the final measurement and was recorded for analysis.

### Statistical Analysis

The Shapiro–Wilk test was then utilized to assess the normality of the outcome data distribution. Accordingly, the Mann–Whitney U test was applied to compare two independent groups with non-normally distributed data, while the Wilcoxon test was used for paired sample data comparisons. Fisher’s exact test was employed to evaluate categorical data. Additionally, the Spearman correlation coefficient was calculated to investigate the relationship between non-normally distributed numeric variables. The interobserver reliability of Cobb angle measurements was assessed by computing the intraclass correlation coefficient (ICC), a reliable metric for determining consistency between assessments made by two examiners. All analyses were performed using IBM SPSS Statistics version 26.0, with a significance level set at α = 0.05 and a confidence level of 95%. Based on the findings of this study and a correlation coefficient value of 0.376, an effect size of 0.613 was determined, which was necessary to achieve 95% statistical power, and this led to a calculated minimum sample size of 37.

## 3. Results

A total of 192 patients under 10 years of age (range: 3–9 years) who underwent adenoidectomy were included in the initial evaluation for spinal attitude assessment roentgenographs which were suitable for the evaluation of scoliotic attitude. A total of 37 patients were included in this study (25 females [67.6%] and 12 males [32.4%]).

The mean age of the included patients in the first evaluation was 5.46 ± 1.86 years and the average follow-up duration was 40.46 ± 23.5 months. At the first evaluation, the Cobb angle for all patients were measured. A total of 15 patients had Cobb angles ≥ 10° with a mean Cobb angle of 12.8° (10° to 17°); the mean Cobb angle for the remaining 22 patients was 6.4° (5° to 9°).

In the second evaluation, clinical investigations—including assessments of pelvic asymmetry and leg length discrepancy—as well as radiological investigations were conducted. The measurement results from the second evaluation were compared for patients with available initial and final assessment data.

Out of the fifteen patients with Cobb angles ≥ 10°, all but three experienced a decrease in their Cobb angles to below 10°, while the remaining three continued to be diagnosed with scoliosis. Among these three scoliosis cases, one patient’s Cobb angle remained unchanged, and while the other two’s values showed a decrease, their Cobb angles remained above 10°.

Among the twenty-two patients with Cobb angles below 10°, one patient exhibited an increase in their Cobb angle to above 10° and was subsequently diagnosed with scoliosis. A reduction in Cobb angle values was observed in sixteen patients within this group, with five patients showing a change of 1°, either as an increase or a decrease.

In the second assessment, which included 37 patients with spinal deformities determined by Cobb angle measurements, several notable findings emerged. One patient was diagnosed with congenital hypothyroidism and their Cobb angle decreased from 14° to 9° during the assessment period. Another patient exhibited a consistent Cobb angle of 11° in both assessments and presented with congenital abnormalities, including the absence of a gallbladder. Additionally, one patient’s Cobb angle increased from 7° to 11° and this individual was found to have a congenital knee dislocation. Among the patients with spinal deformities measuring below 10°, two had noteworthy medical histories: one had a family history of scoliosis, while the other had a history of epilepsy. These findings highlight the diverse clinical characteristics associated with spinal deformities in this cohort and underscore the importance of comprehensive assessment in understanding the underlying factors contributing to these conditions.

The ICC for the interobserver reliability of measuring a Cobb angle was 0.938. This highlights a remarkable level of concordance, precision, and accuracy in the assessments performed by the two observers. This substantial ICC value portrays a commendable consistency in the evaluations of spinal curvature among the patients.

**Figure 1:**
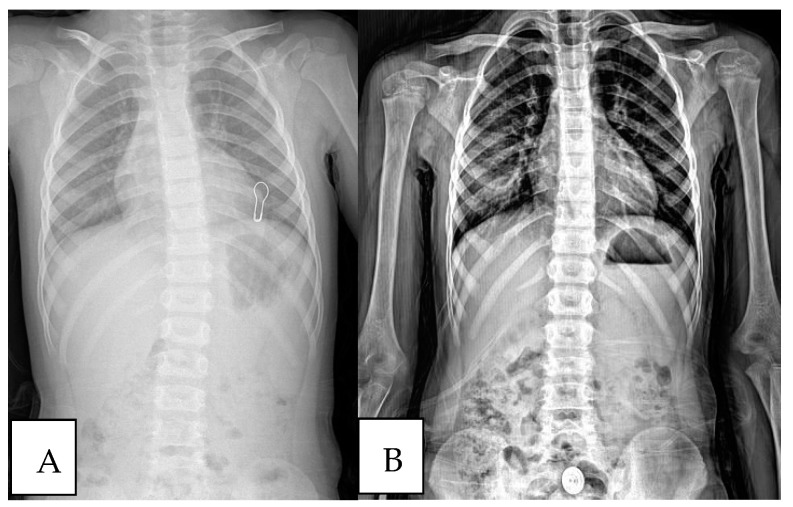
(**A**) Chest roentgenograph of a patient with a scoliotic attitude detected during preoperative preparation. (**B**) Post-adenoidectomy scoliosis roentgenograph of the same patient.

**Figure 2:**
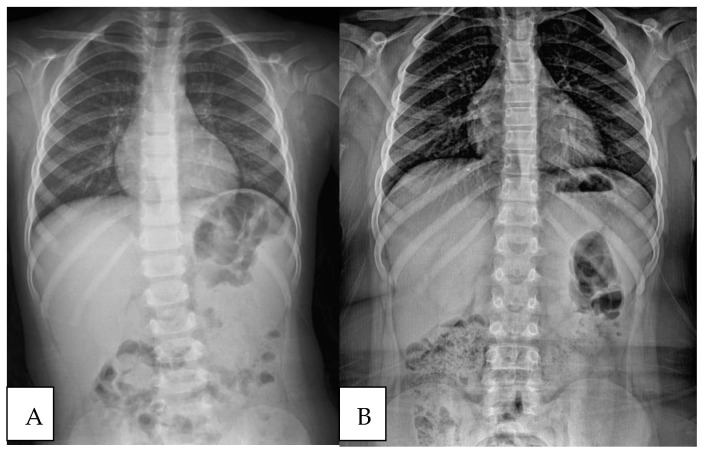
(**A**) Chest roentgenograph of another patient with a scoliotic attitude detected during preoperative preparation. (**B**) Post-adenoidectomy scoliosis roentgenograph of the same patient.

**Figure 3:**
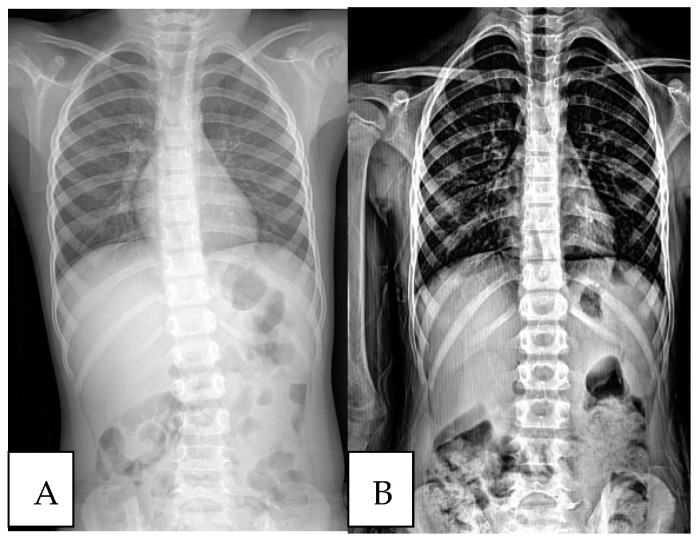
(**A**) Chest roentgenograph of another patient with a scoliotic attitude detected during preoperative preparation. (**B**) Post-adenoidectomy scoliosis roentgenograph of the same patient.

In Figure 1, Figure 2 and Figure 3, three different patient’s radiological investigations before and after their adenoidectomy operations are seen, respectively.

The analysis results regarding the statistical evaluation of the change between preoperative Cobb angles and postoperative Cobb angles are presented in Table 1.

According to Table 1, there was a statistically significant difference between the preoperative Cobb angle and the Cobb angle measured postoperatively in patients, with the preoperative Cobb angle being significantly higher than the postoperative one (*p* < 0.001). In individuals with Cobb angles < 10°, there was a statistically significant difference between the preoperative and postoperative degrees (*p* < 0.001). The preoperative Cobb angle was significantly higher than the postoperative one. Similarly, in individuals with Cobb angles ≥ 10°, there was a statistically significant difference between the preoperative and postoperative degrees (*p* < 0.001).

The analysis results assessing whether the postoperative Cobb angle measurements differed based on age, gender, and follow-up duration are provided in Table 2.

Upon examining Table 2, it is evident that the postoperative Cobb angle had a statistically significant, positive, and moderate correlation with age (*p* = 0.022). Therefore, as age increased, the measured Cobb angle after adenoidectomy also significantly increased. However, the postoperative Cobb angle did not exhibit a statistically significant difference with respect to gender and follow-up duration (*p* = 0.936 and *p* = 0.320, respectively).

The analysis results evaluating whether there were differences in the postoperative Cobb angle based on age, gender, and follow-up duration in patients with preoperative Cobb angles below 10° are presented in Table 3.

According to Table 3, there were no statistically significant differences observed in postoperative Cobb angles with regards to age, gender, and follow-up duration (*p* > 0.05).

Similarly, the analysis results pertaining to patients with preoperative Cobb angles of 10° or higher, which evaluated differences in postoperative Cobb angle based on age, gender, and follow-up duration, are shown in Table 4.

As indicated in Table 4, there were no statistically significant differences observed in postoperative Cobb angles with respect to age, gender, and follow-up duration (*p* > 0.05).

Lastly, the analysis results investigating differences in age, follow-up duration, and gender between patients with preoperative Cobb angles of 10° and above versus those with Cobb angles below 10° are presented in Table 5.

According to Table 5, patients with a preoperative Cobb angle of 10° and above did not exhibit statistically significant differences in terms of age, gender, and follow-up duration when compared to patients with a Cobb angle below 10° (*p* > 0.05).

## 4. Discussion

Adenoidectomy is the primary surgical approach for treating OSA, either with or without AT [21,22,31]. Adenoidectomy presents fewer complications compared to AT and allows reduced postoperative discomfort, decreased risk of dehydration, minimized postoperative bleeding, and fewer or shorter hospital stays, resulting in considerable cost savings. Therefore, the preferred choice of treatment for OSA is often an adenoidectomy [21,22]. In cases of adenoid hypertrophy, an increase in HIF and oxidative stress has been observed under hypoxic conditions [24,25]. Pioloux et al. [32] demonstrated the relationship between oxidative stress and HIF, and stated that hypoxia triggers oxidative stress through the excessive generation of reactive oxygen species. It has been shown that elevated HIF levels after adenoidectomy decrease significantly within four weeks [24]. Lin et al. [33] demonstrated the selective inhibition of the HIF signaling pathway in human tonsil epithelial cells using molecular effectors, suggesting a promising therapeutic strategy for addressing hypoxia-induced sleep and breathing disorders caused by adenoid hypertrophy.

Rivard et al. [13] conducted one of the initial studies demonstrating the impact of hypoxia in scoliosis using mice as subjects; they induced congenital vertebral malformations, which closely resembled those observed in humans, using hypoxia as a teratogenic agent. Suvarnan et al. [19] investigated the hypoxic response pathway by exposing tissues to hypoxia, and established the critical involvement of HIF-1 and HIF-2 [14,32]. They found that hypoxia leads to the downregulation of the PITX1 gene in the cellular culture of human osteoblasts, proposing its role in this process [19].

It is known that the PITX1 gene is associated with various bone-related diseases [34,35]. Fendri et al. [34] demonstrated that PITX1 gene expression is significantly lower in patients with AIS. The relationship between hypoxia-induced HIF and the PITX1 gene has been established [36], highlighting the importance of this gene. Specifically, PITX1 is crucial in regulating cartilage development, bone development, and muscle shaping [35]. In its absence, morphological changes to bone structure have been observed [37].

It has been clearly demonstrated that DNA methylation plays a significant role in reduced PITX1 gene expression due to relatively high promoter region methylation, which may be associated with the etiology of AIS [18,19]. To further investigate the specific impact of abnormal PITX1 methylation on the clinical and biological characteristics of AIS patients, the age, gender, and Cobb angles were compared between AIS patients with positive and negative PITX1 methylation. These results revealed a significant difference in the age and Cobb angle of the patients between the two groups [18].

Tam et al. and Suvarnan et al. also revealed that the overexpression of HIFs could serve as an indicator for assessing the severity and progression of idiopathic scoliosis [14,19]. Interestingly, HIF-2 was identified as having greater potency than HIF-1 in activating PITX1 transcription, and partial inactivation of the PITX1 gene resulted in severe spinal deformities [19]. On the other hand, Tam et al. [14] suggested that the dysregulation of HIF, a transcription factor that responds to hypoxia (a decrease in oxygen availability in the cellular environment), might be a risk factor for idiopathic scoliosis. In this study, a significant decrease in the Cobb angle was observed following adenoidectomy, which may be attributed to the postoperative reduction in hypoxic effects [24]. However, further research is warranted to evaluate hypoxia as a risk factor for scoliosis findings.

Additionally, it has been demonstrated that delayed surgical indications in OSA may diminish its impact on the patient’s recovery; consequently, surgery before the age of seven years is often recommended for residual OSA [21,22,38]. The duration of hypoxia exposure is another variable. In the present study, we observed that the period of recovery following both short- and long-term intermittent exposure to hypoxia aligned with the duration of its impact. While complete recovery may occur after short-term exposure, persistent abnormalities prevail following long-term exposure, even after the removal of the causal factor [20]. We also observed that the patient’s age at the time of adenoidectomy significantly affected the change in the Cobb angle, and that surgery being performed at a younger age led to a greater change in the Cobb angle.

Previous studies have suggested that adenoid size increases during childhood, usually reaching its maximal size by the age of six or seven years, before spontaneously regressing during adolescence [39]. The spontaneous regression of scoliosis is a known phenomenon [40,41]. The majority of studies have focused on the reasons behind scoliosis progression [42,43]. It is essential to understand how elevated OSA (Obstructive Sleep Apnea) caused by enlarged adenoids is alleviated once the condition is corrected, and to what extent this improvement occurs. It is known that in men, correcting hypoxia associated with adenoids can reverse many effects, but it should not be overlooked that when hypoxia persists for an extended period, these effects may not be observed. The current priority is to understand the reason behind and the extent of spontaneous regression of scoliosis. Moreover, idiopathic scoliosis, which develops in infancy, is resolved spontaneously in 80–90% of patients [44].

Soucacos et al. [41] reported that a complete resolution of curvature was seen in 9.5% of their patients and >27.4% of patients exhibited a spontaneous decrease in the magnitude of the curve of at least 10°. Likewise, Brooks et al. [45] noted a spontaneous improvement in 22% of patients. Modi et al. [40] describes ‘spontaneous regression’ identified in immature children as a ‘balancing or tuning mechanism’ occurring within the spinal column, which highlights its role in achieving balance and natural correction. Hawes et al. [8] introduced the vicious cycle model, which suggests that spinal curvature can be diagnosed at an early stage and can be corrected when subjected to asymmetric loading for an extended period. There is a lack of substantive evidence corroborating the concept and underlying causes of spontaneous regression in the context of scoliosis. We contend that our study provides novel insights into the phenomenon of spontaneous regression in spinal curvature, which, contrary to its designation, is not a spontaneous event but rather a gradual process.

We maintain that, as demonstrated above, structural improvements will occur with the resolution of conditions frequently causing hypoxia in children before they become irreversible [46], and that this has an impact on the Cobb angle. Our study provides evidence supporting this effect [20].

The Cobb angle data analyzed in our initial assessment were obtained from preoperative chest radiographs. Although Oh et al. [29] reported that chest radiography has limited value due to the omission of lumbar curvature evaluations, they also noted that thoracic curvatures were identified with a sensitivity of 93.94%, whereas the identification of lumbar curvatures was less consistent. Similarly, Kockara et al. [28] described chest radiography as a valuable method for monitoring asymptomatic thoracic scoliosis, emphasizing that it accurately identified thoracic curvatures. In our study, only preoperative standing radiographs suitable for scoliosis assessment were included. This was achieved by utilizing data that accounted for potential imaging errors resulting from discrepancies between the recommended imaging field and the actual imaging field during pediatric chest radiography [26].

The Cobb angle is a gold standard measurement for scoliosis evaluation, and we used this for our initial assessment. A Cobb angle exceeding 10° is universally accepted as the diagnostic criterion for scoliosis and it holds significant importance in assessing the severity of scoliosis [47,48]. Although deficiency in the initial clinical data was supplemented by the second assessment, it is worth recognizing that a strong correlation exists between clinical and radiological parameters, illustrating a parallel relationship between both in describing the extent of the deformity. It is known that the degree of curvature, as indicated by the Cobb angle, aligns with the prominence of surface deformity [43]. In our study, the Cobb angles measured for all patients ranged from 5° to 17°, which suggests that these measurements can compensate for the lack of available clinical data, and that it cannot be disregarded as a measure of spinal curvature. This range is unlikely to substantially influence patient diagnoses.

This study has certain limitations. First, the absence of lateral vertebral column radiographs in the initial evaluation precluded the evaluation of spinal deformities in the sagittal plane; therefore, we were unable to achieve a comprehensive understanding of the three-dimensional distortion of the spine. Second, we used a retrospective study design, which obscured our ability to obtain detailed clinical data about patients during the initial evaluation, thus limiting the depth of insight into potential influencing factors. Another limitation of this study is the relatively small sample size. While our findings provide valuable insights into the potential association between scoliosis and hypoxia, the small cohort limits the generalizability of the results. As one of the few studies investigating this relationship, this research serves as a preliminary step in understanding the impact of eliminating hypoxic factors on scoliosis outcomes. Given the preliminary nature of this study, future research with larger sample sizes is necessary to validate these findings and further explore the mechanisms involved. Expanding the cohort in subsequent studies will ensure the robustness of the observed associations and provide a clearer picture of the clinical implications. Prospective studies involving a larger cohort of patients are warranted to gain a more comprehensive perspective and shed light on the interaction between adenoidectomy, hypoxia, and scoliosis regression.

## 5. Conclusions

This study indicates a significant reduction in the Cobb angle following adenoidectomy, suggesting a possible link between decreased hypoxia-related effects and improved lumbar curvature, with a notable correlation between patient age at surgery and postoperative Cobb angle changes. These findings provide new insights into the interplay between adenoidectomy, hypoxia, and scoliotic regression in pediatric patients, highlighting the need for further research to explore these relationships and refine clinical strategies for scoliosis management.

## Figures and Tables

**Table 1:** Examination of Cobb angle changes preoperatively and postoperatively.

	Initial Assessment	Final Assessment	*p*-Value *
**Cobb Angle**	7 (5–17)	3 (0–12)	**<0.001**
**Cobb Angle < 10°**	6.25 (5–9)	0.50 (0–11)	**<0.001**
**Cobb Angle ≥ 10°**	12 (10–17)	5 (0–12)	**<0.001**

Data are presented as the median (min–max). * These are *p*-values from the Wilcoxon test.

**Table 2:** The final assessment of the Cobb angle evaluation according to age, gender, and follow-up duration.

	Final Assessment Cobb Angle	*p*-Value
**Age**	r = 0.376	0.022 **
**Gender**		
**Female**	3 (0–11)	0.936 ***
**Male**	3 (0–12)	
**Follow-up time (months)**	r = −0.168	0.320 **

Data are presented as the median (min–max). ** These are *p*-values from the Spearman’s Correlation Coefficient. *** This is the *p*-value from the Mann–Whitney U test.

**Table 3:** The evaluation of the final assessment Cobb angles according to age, gender, and follow-up duration in individuals with an initial Cobb angle < 10°.

	Final Assessment Cobb Angle	*p*-Value
**Age**	r = 0.270	0.224 **
**Gender**		
**Female**	0 (0–11)	0.821 ***
**Male**	1.50 (0–7)	
**Follow-up time (months)**	r = −0.044	0.845 **

Data are presented as the median (min–max). ** These are *p*-values from the Spearman’s Correlation Coefficient. *** This is the *p*-value from the Mann–Whitney U test.

**Table 4:** The evaluation of the final assessment Cobb angles according to age, gender, and follow-up duration in individuals with an initial Cobb angle ≥ 10°.

	Final Assessment Cobb Angle	*p*-Value
**Age**	r = 0.465	0.081 **
**Gender**		
**Female**	4 (0–11)	0.114 ***
**Male**	10.50 (9–12)	
**Follow-up time (months)**	r = −0.231	0.407 **

Data are presented as the median (min–max). ** These are *p*-values from the Spearman’s Correlation Coefficient. *** This is the *p*-value from the Mann–Whitney U test.

**Table 5:** Evaluation of age, gender, and follow-up duration in individuals with Cobb Angles ≥ 10° and those with Cobb Angles < 10° during initial assessment.

	Cobb Angle < 10	Cobb Angle ≥ 10	*p*-Value
**Age**	6 (3–8)	6 (3–8)	0.915 ***
**Gender**			
**Female**	12 (54.5%)	13 (86.7%)	0.073 ^&^
**Male**	10 (45.5%)	2 (13.3%)	
**Follow-up time (months)**	47.50 (7–70)	43 (6–78)	0.366 ***

Numerical data are presented as the median (min–max), while categorical data are presented as a frequency (percentage). *** These are *p*-values from the Mann–Whitney U test. ^&^ This is the *p*-value from the Fisher’s exact test.

## Data Availability

The data and materials generated/analyzed in the present study are available from the corresponding author upon request due to privacy.

## Abbreviations

OSA	Obstructive Sleep Apnea
EOS	Early-onset scoliosis
HIF	Hypoxia-inducible factor
AT	Adenotonsillectomy
ICC	Intraclass correlation coefficients

## References

[1] Zaina F., Donzelli S., Negrini S. (2023). Idiopathic Scoliosis: Novel Challenges for Researchers and Clinicians. Children.

[2] Konieczny M.R., Senyurt H., Krauspe R. (2013). Epidemiology of Adolescent Idiopathic Scoliosis. J. Child. Orthop..

[3] Horng M.-H., Kuok C.-P., Fu M.-J., Lin C.-J., Sun Y.-N. (2019). Cobb Angle Measurement of Spine from X-ray Images Using Convolutional Neural Network. Comput. Math. Methods Med..

[4] Bernstein P., Metzler J., Weinzierl M., Seifert C., Kisel W., Wacker M. (2020). Radiographic Scoliosis Angle Estimation: Spline-Based Measurement Reveals Superior Reliability Compared to Traditional COBB Method. Eur. Spine J..

[5] Fadzan M., Bettany-Saltikov J. (2017). Etiological Theories of Adolescent Idiopathic Scoliosis: Past and Present. Open Orthop. J..

[6] Gorman K.B., Julien C., Moreau A. (2012). The Genetic Epidemiology of Idiopathic Scoliosis. Eur. Spine J..

[7] Dayer R., Haumont T., Belaieff W., Lascombes P. (2013). Idiopathic Scoliosis: Etiological Concepts and Hypotheses. J. Child. Orthop..

[8] Hawes M.C., O’Brien J.P. (2006). The Transformation of Spinal Curvature into Spinal Deformity: Pathological Processes and Implications for Treatment. Scoliosis.

[9] Burwell R.G., Clark E.M., Dangerfield P.H., Moulton A. (2016). Adolescent Idiopathic Scoliosis (AIS): A Multifactorial Cascade Concept for Pathogenesis and Embryonic Origin. Scoliosis Spinal Disord..

[10] Shivan M., Tambe A.D., Millner P., Tsirikos A.I. (2022). Adolescent Idiopathic Scoliosis. Bone Jt. J..

[11] Erol B., Kusumi K., Lou J., Dormans J. (2002). Etiology of Congenital Scoliosis. Univ. Pennsylvania Orthop. J..

[12] Farley F.A., Loder R.T., Nolan B.T., Dillon M.T., Frankenburg E.P., Kaciroti N.A., Miller J.D., Goldstein S.A., Hensinger R.N. (2001). Mouse Model for Thoracic Congenital Scoliosis. J. Pediatr. Orthop..

[13] Rivard C.H. (1986). Effects of Hypoxia on the Embryogenesis of Congenital Vertebral Malformations in the Mouse. Clin. Orthop. Relat. Res..

[14] Tam W.K., Cheung J.P.Y., Koljonen P.A., Kwan K.Y.H., Cheung K.M.C., Leung V.Y.L. (2022). Slow Twitch Paraspinal Muscle Dysregulation in Adolescent Idiopathic Scoliosis Exhibiting HIF-2α Misexpression. JOR Spıne.

[15] Liu S.-R., Ren D., Wu H.-T., Yao S.-Q., Song Z.-H., Geng L.-D., Wang P.-C. (2022). Reparative Effects of Chronic Intermittent Hypobaric Hypoxia Pre-Treatment on Intervertebral Disc Degeneration in Rats. Mol. Med. Rep..

[16] Lafont J.E. (2010). Lack of Oxygen in Articular Cartilage: Consequences for Chondrocyte Biology. Int. J. Exp. Pathol..

[17] Li J., Tang M., Yang G., Wang L., Gao Q., Zhang H. (2019). Muscle Injury Associated Elevated Oxidative Stress and Abnormal Myogenesis in Patients with Idiopathic Scoliosis. Int. J. Biol. Sci..

[18] Shi B., Xu L., Mao S., Xu L., Liu Z., Sun X., Zhu Z., Qiu Y. (2018). Abnormal PITX1 Gene Methylation in Adolescent Idiopathic Scoliosis: A Pilot Study. BMC Musculoskelet. Disord..

[19] Suvarnan L. (2010). The Role of Transcription Factor Pitx1 and Its Regulation by Hypoxia in Adolescent Idiopathic Scoliosis. Master’s Thesis.

[20] Nanduri J., Semenza G.L., Prabhakar N.R. (2017). Epigenetic Changes by DNA Methylation in Chronic and Intermittent Hypoxia. Am. J. Physiol.-Lung Cell. Mol. Physiol..

[21] Domany K.A., Dana E., Tauman R., Gut G., Greenfeld M., Yakir B.-E., Sivan Y. (2016). Adenoidectomy for Obstructive Sleep Apnea in Children. J. Clin. Sleep Med..

[22] Schupper A.J., Nation J., Pransky S. (2018). Adenoidectomy in Children: What Is the Evidence and What Is Its Role?. Curr. Otorhinolaryngol. Rep..

[23] Zaffanello M., Ersu R.H., Nosetti L., Beretta G., Agosti M., Piacentini G. (2024). Cardiac Implications of Adenotonsillar Hypertrophy and Obstructive Sleep Apnea in Pediatric Patients: A Comprehensive Systematic Review. Children.

[24] Abuhandan M., Bozkuş F., Demir N., Eren E., Koca B., Kadir Guler O., Selek S. (2013). The Preoperative and Postoperative Oxidative Status of Children with Chronic Adenotonsillar Hypertrophy. La Clin. Ter..

[25] Yucel K., Aydin I., Erdem S.S. (2020). Hypoxia Induced Factor-1α Levels in Patients Undergoing Adenoidectomy. Scand. J. Clin. Lab. Investig..

[26] Mahomed N., Manduku V., Hlabangana L., Gong W., Moore D., Fancourt N., Madhi S. (2023). Evaluation of paediatric chest X-ray quality in the Pneumonia Aetiology Research for Child Health (PERCH Study), a prospective international multicentre study in 7 developing countries. Wits J. Clin. Med..

[27] Pan X.-X., Huang C.-A., Lin J.-L., Zhang Z.-J., Shi Y.-F., Chen B.-D., Zhang H.-W., Dai Z.-Y., Yu X.-P., Wang X.-Y. (2020). Prevalence of the Thoracic Scoliosis in Children and Adolescents Candidates for Strabismus Surgery: Results from a 1935-Patient Cross-Sectional Study in China. Eur. Spine J..

[28] Kockara N., Ucpunar H. (2019). The Value of Standard Chest Radiography in the Diagnosis of Scoliosis. Ann. Med. Res..

[29] Oh C.H., Kim C.G., Lee M.S., Yoon S.H., Park H.-C., Park C.O. (2012). Usefulness of Chest Radiographs for Scoliosis Screening: A Comparison with Thoraco-Lumbar Standing Radiographs. Yonsei Med. J..

[30] Parr A., Askin G. (2020). Paediatric Scoliosis: Update on Assessment and Treatment. Aust. J. Gen. Pract..

[31] Wang H., Qiao X., Qi S., Zhang X., Li S. (2021). Effect of Adenoid Hypertrophy on the Upper Airway and Craniomaxillofacial Region. Transl. Pediatr..

[32] Pialoux V., Mounier R., Brown A.D., Steinback C.D., Rawling J.M., Poulin M.J. (2009). Relationship between Oxidative Stress and HIF-1α MRNA during Sustained Hypoxia in Humans. Free Radic. Biol. Med..

[33] Lin Y., Wang M., Xiao Z., Jiang Z. (2021). Hypoxia Activates SUMO-1-HIF-1α Signaling Pathway to Upregulate Pro-Inflammatory Cytokines and Permeability in Human Tonsil Epithelial Cells. Life Sci..

[34] Fendri K., Patten S.A., Kaufman G.N., Zaouter C., Parent S., Grimard G., Edery P., Moldovan F. (2013). Microarray Expression Profiling Identifies Genes with Altered Expression in Adolescent Idiopathic Scoliosis. Eur. Spine J..

[35] Zhao J., Xu Y. (2023). PITX1 Plays Essential Functions in Cancer. Front. Oncol..

[36] Mudie S., Bandarra D., Batie M., Biddlestone J., Moniz S., Ortmann B., Shmakova A., Rocha S. (2014). PITX1, a Specificity Determinant in the HIF-1α-Mediated Transcriptional Response to Hypoxia. Cell Cycle.

[37] Wang J.S., Infante C.R., Park S., Menke D.B. (2018). PITX1 Promotes Chondrogenesis and Myogenesis in Mouse Hindlimbs through Conserved Regulatory Targets. Dev. Biol..

[38] Imanguli M., Ulualp S.O. (2016). Risk Factors for Residual Obstructive Sleep Apnea after Adenotonsillectomy in Children. Laryngoscope.

[39] Geiger Z., Gupta N. Adenoid Hypertrophy. https://www.ncbi.nlm.nih.gov/books/NBK536984/.

[40] Modi H.N., Suh S.W., Yang J.H., Hong J.Y., Venkatesh K., Muzaffar N. (2010). Spontaneous Regression of Curve in Immature Idiopathic Scoliosis—Does Spinal Column Play a Role to Balance? An Observation with Literature Review. J. Orthop. Surg. Res..

[41] Soucacos P.N., Zacharis K., Gelalis J., Soultanis K., Kalos N., Beris A., Xenakis T., Johnson E.O. (1998). Assessment of Curve Progression in Idiopathic Scoliosis. Eur. Spine J..

[42] Noshchenko A. (2015). Predictors of Spine Deformity Progression in Adolescent Idiopathic Scoliosis: A Systematic Review with Meta-Analysis. World J. Orthop..

[43] Lonstein J.E., Carlson J.M. (1984). The Prediction of Curve Progression in Untreated Idiopathic Scoliosis during Growth. J. Bone Jt. Surg..

[44] Karpe P., Killen M.-C., Fender D. (2020). Scoliosis in Childhood. Surgery.

[45] Brooks H.L., Azen S.P., Gerberg E., Brooks R., Chan L. (1975). Scoliosis: A prospective epidemiological study. J. Bone Jt. Surg..

[46] Saran S., Saccomanno S., Viti S., Mastrapasqua R.F., Viti G., Giannotta N., Fioretti P., Lorenzini E., Raffaelli L., Levrini L. (2024). Analysis of General Knowledge on Obstructive Sleep Apnea Syndrome (OSAS) among Italian Pediatricians. Children.

[47] Kotwicki T., Kinel E., Stryla W., Szulc A. (2007). Discrepancy in Clinical versus Radiological Parameters Describing Deformity due to Brace Treatment for Moderate Idiopathic Scoliosis. Scoliosis.

[48] Su X., Dong R., Wen Z., Liu Y. (2022). Reliability and Validity of Scoliosis Measurements Obtained with Surface Topography Techniques: A Systematic Review. J. Clin. Med..

